# Effects of relaxation training after volleyball exercise on blood lactate concentration, muscle hardness, and heart rate

**DOI:** 10.3389/fphys.2025.1644183

**Published:** 2025-10-30

**Authors:** Yonghong Bian

**Affiliations:** College of Physical Education of Inner Mongolia Minzu University, Tongliao, China

**Keywords:** volleyball, recovery of function, relaxation therapy, lactate, heart rate, muscle tonus

## Abstract

**Background:**

Volleyball recovery optimisation is not a well-studied topic although recovery is physiologically demanding. This experiment was conducted to determine the effectiveness of guided relaxation training over passive rest based on biochemical, mechanical and autonomic recovery measures.

**Methods:**

A total of 600 adolescent volleyball athletes (16.8 ± 1.1 years) were recruited across four training centres in China. Participants completed both relaxation and passive recovery conditions in a randomised crossover design, with a one-week washout between sessions. Blood lactate, muscle hardness (via Myoton), heart rate recovery at 60 s (HRR60s), total quality recovery (TQR), and soreness using a visual analogue scale (VAS) were measured at baseline, T1 (immediate post), T2 (15 min), and T3 (30 min). Nonparametric comparisons (Mann–Whitney U test) and an aligned rank transform analysis of variance (ANOVA) were used for between-group and within-group analyses, respectively.

**Results:**

At T3, mean lactate was significantly lower in the relaxation group (2.02 ± 0.72 mmol/L) than passive rest (2.76 ± 0.81 mmol/L; U = 34,011, p = 0.001). Muscle hardness reduction was greater in the relaxation group (−18.04 ± 7.12 N/m) compared to passive rest (−11.12 ± 6.87 N/m; U = 36,291, p = 0.002). HRR60s improved more markedly in the relaxation group (+31.84 ± 6.72 bpm vs. +26.91 ± 7.20 bpm; U = 33,712, p = 0.001). TQR scores were higher (15.9 ± 1.6 vs. 14.7 ± 1.9; U = 32,598, p = 0.001) and VAS soreness lower (2.4 ± 1.2 vs. 3.1 ± 1.4; U = 35,811, p = 0.001) under relaxation.

**Conclusion:**

Relaxation training enhances multidimensional recovery outcomes in competitive volleyball athletes.

## 1 Introduction

Recovery optimisation in volleyball remains insufficiently addressed despite the high physiological and mechanical demands documented in recent applied exercise research (Gao et al., 2024). Match-and-drill formats commonly drive sustained internal load and delayed restitution in collegiate populations (Jeong et al., 2024). Short work-to-rest cycles and repeated jumps further complicate post-exercise restoration in youth and sub-elite cohorts (Li et al., 2024). Progressive muscle relaxation, diaphragmatic breathing and guided meditation are combined with relaxation training to enhance parasympathetic and decrease sympathetic activities following exertion (Petviset et al., 2025). Changes in biomarker during relaxation are not limited to mental states but also involve the level of lactate in the blood and the tone of neuromuscular management under the field conditions (Rudics et al., 2025). The unique monitoring of volleyball suggests that frequent levels of lactates in explosive stages are high, which highlights the importance of recovery tactics that would hasten the process of clearance (Pisa et al., 2025). The same session analysis results in adolescents indicate lactate peaks that are also in line with high anaerobic contribution (Veiga et al., 2025).

Muscle hardness, quantified with handheld devices, is an objective indicator of localised fatigue and is poorly responsive to simple passive approaches such as static stretching (Takeuchi et al., 2024). Neural and muscular coupling exhibits measurable drift with accumulating fatigue, suggesting the value of paired cardiac and mechanical surveillance (Langan, 2025). Seasonal monitoring in adolescent female athletes shows immune-linked vulnerability during periods of under-recovery (Yasuda, 2025). Motivational decrements also accompany chronically elevated load and insufficient restoration in competitive programs (Saki, 2025). Field-irrelevant biochemical aids do not translate well into routine practice in team environments (Meng and Zhang, 2025).

Lactate clearance rate (LCR) is a practical index of metabolic resilience and can inform readiness during repeated stress exposures (Yu L. et al., 2025). Post-match data in elite female volleyball players indicate persistent cardiac and metabolic strain that standard cooldowns may not resolve (Akarçeşme et al., 2022). Already, conceptual suggestions are set to use tri-marker tracking based on the heart rate recovery, lactate, and mechanical stiffness, even though there are few empirical tests (Widowati et al., 2025). Cross-modal conditioning experiments record cardiovascular, but do not measure lactate or stiffness to permit integrative inference to volleyball (Cuesta-Vargas et al., 2009). Volleyball, post-training vagal withdrawal and longer sympathetic dominance Heart rate (HR) and heart rate variability (HRV) syntheses are always characterized by the fact that autonomic-targeted recovery is of great importance (Cardoso et al., 2022). The biological maturation attenuates HR recovery patterns in adolescent athletes, which implies stratified interpretation of the autonomic markers (de Almeida-Neto et al., 2025). High-intensity exercise is associated with molecular stories concerning neurotoxic signalling orchestras that could also interrelate with perceived recovery and central control (Li et al., 2025).

Non-volleyball evidence indicates that basic-after exercise modalities have the potential to change cardiovascular condition to the recovery state but not always lactate kinetics is detected (Keller and Chilibeck, 2025). The fitness level also influences lactate decrease during resistance events, which indicates personal factors influencing the recovery of the metabolism (Del-Cuerpo et al., 2025). Following high-intensity interval training, brief yoga enhances HRV and breathing economy, suggesting a parasympathetic pathway that can be used in addition to metabolic recovery and mechanical recovery in sport (Petviset et al., 2025). Monitoring of body-composition at camps confirms that macro-observable changes are not always concomitants of acute recovery condition, and there is a strong rationale to use direct physiological indicators in volleyball (Podstawski et al., 2025).

Sport-specific fatigue impairs neurocognitive performance, but most protocols do not include concomitant physiological monitoring, which limits a mechanistic explanation of the impact of recovery (Yu Y. et al., 2025). Technical decision-making results with adolescent players can be enhanced by guided imagery, which implies that readiness can be facilitated using behavioural methods even when physiological markers have not been measured (Fortes et al., 2020). Breathing interventions improve HRV and interoceptive control, which is aligned with central autonomic processes, which might affect muscle tone and perceived recovery (Sakaj, 2025).

**FIGURE 1:**
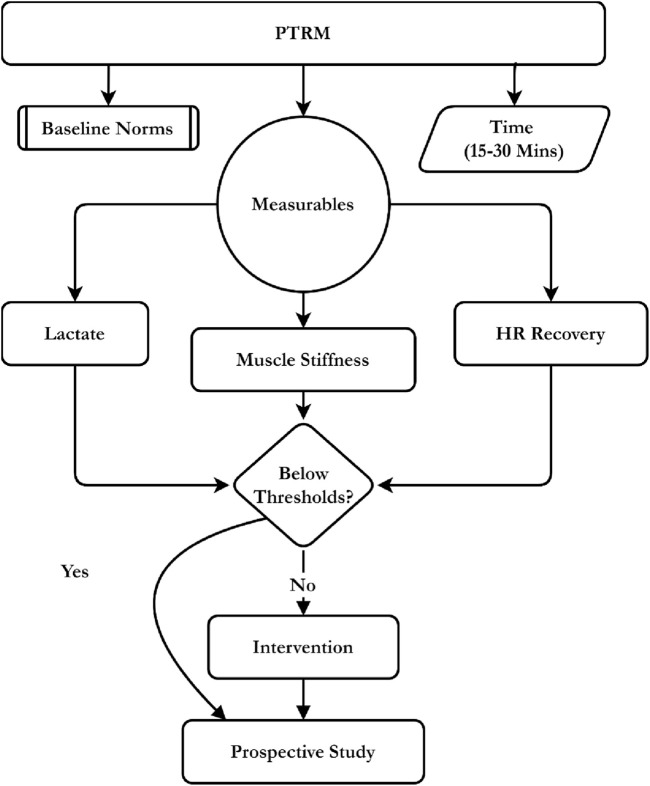
Conceptual framework of the PTRM integrating lactate, stiffness, and HR recovery for intervention.

This study uses the Prospective Tri-Marker Recovery Model (PTRM) to address the biochemical, mechanical, and autonomic indices following volleyball exercise. The model quantifies blood lactate concentration, muscle hardness and heart rate recovery in one post-exercise wind-up utilizing field-portable analysers, mechanical probes, and heart rate monitors to generate a practical, sport-specific portrait (theoretical summary illustrated in Figure 1).

The present study aimed to:

1. Determine the influences of a 15 min guided relaxation regimen on post-volleyball blood lactate levels, muscle hardness, and recovery of HR.
2. Compare the results of passive recovery to relative efficacy.
3. Test the possibility of using the combination of these markers as a multidimensional model of quantifying post-exercise recovery.

The hypothesised effects of relaxation training included larger changes in blood lactate and muscle hardness and accelerated HR recovery than positive changes in passive rest, which would eventually support the PTRM as a holistic model to individualised recovery evaluation of volleyball.

## 2 Materials and methods

### 2.1 Research design

This study was a randomised crossover study that follows the Strengthening the Reporting of Observational Studies in Epidemiology (STROBE) recommendations. Everyone took both of the recovery conditions relaxation training and passive recovery. The randomisation was done and the order was separated by a 7-day washout. Sessions were introduced by regular volleyball-specific exercise and then the recovery procedure. The measure of recovery markers was done at four set post-exercise time points. The within subject design allowed comparing the responses directly across the two conditions and this controlled the inter-individual variability.

All procedures were done in the Human Performance Laboratory, Faculty of Sports Science, China. The meetings were arranged between 4:00 p.m. and 6:00 p. m. to manage circadian variation. The conditions were kept as follows: 22 °C–1 °C temperature and 55–3 percent relative humidity (using an HTC-2 Thermo-Hygrometer, China) ambient sound reading of less than 40 d B (using a Benetech GM1357 Sound Meter, China) and 300 lux illumination (using an LED source, using an Extech SDL400, United States).

### 2.2 Participants

#### 2.2.1 Inclusion and exclusion criteria

**FIGURE 2:**
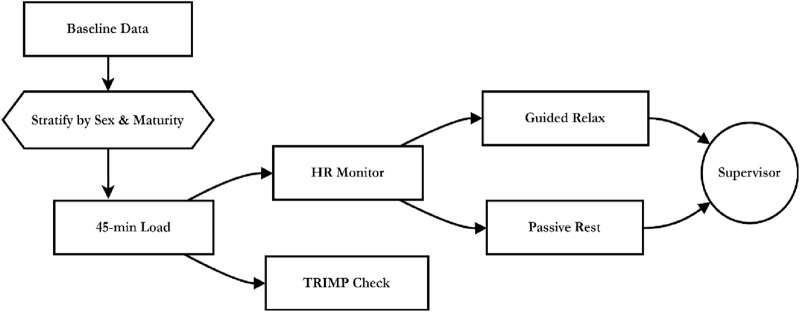
Procedural flow of stratified volleyball load, HR monitoring, and recovery under supervisor oversight.

Participants were eligible if they met the following criteria: (i) age between 16.0 and 25.9 years; (ii) minimum of three consecutive years of competitive volleyball training history; (iii) active engagement in structured training ≥3 sessions per week; (iv) resting heart rate between 50 and 90 beats per minute measured after 10 min supine rest; (v) body mass index between 18.5 and 26.5 kg/m^2^ measured using standardized digital anthropometry; and (vi) biological maturity within the range of −2.0 to +2.0 years from peak height velocity, estimated using the Mirwald equation (Mirwald et al., 2002) (Figure 2):

$$\text{Maturity Offset}=-9.236+0.0002708\times \left(\text{Leg Length}\times \text{Sitting Height}\right)-0.001663\times \left(\text{Age}\times \text{Leg Length}\right)+0.007216\times \left(\text{Age}\times \text{Sitting Height}\right)+0.02292\times \left(\text{Weight}/\text{Height Ratio}\right)$$

where *Leg Length* = standing height–sitting height (cm), *Sitting Height* = cm, *Age* = chronological age in years, *Weight/Height Ratio* = body mass (kg) ÷ standing height (cm).

All anthropometric data were obtained by two trained assessors using International Society for the Advancement of Kinanthropometry (ISAK) Level 1 standards. Standing and sitting heights were measured to the nearest 0.1 cm with a Seca 213 stadiometer (Germany), and body mass to the nearest 0.1 kg with a Tanita BC-601 digital scale (Japan). Each measure was repeated twice, with a third taken if variation exceeded 0.3 cm or 0.2 kg; the mean of consistent readings was used. Participants were barefoot and wore light athletic clothing. Calculations were performed immediately in Microsoft Excel using verified templates to minimise transcription errors. The resulting maturity offset values allowed classification within ±2.0 years of PHV across cohorts.

Participants were excluded if they met any of the following: (i) history or current diagnosis of cardiovascular conditions such as arrhythmia or hypertension; (ii) metabolic or endocrine disorders including diabetes mellitus or thyroid dysfunction; (iii) neurological conditions including epilepsy or autonomic instability; (iv) use of medications affecting heart rate, vascular tone, or skeletal muscle physiology within 30 days; (v) musculoskeletal injury occurring within 28 days before testing; (vi) participation in recovery-enhancing modalities such as cryotherapy, massage, compression garments, or ergogenic aids within 48 h pre-test; or (vii) clinical indicators of overreaching including resting heart rate >95 bpm, persistent fatigue exceeding 5 days, or sleep duration <6 h per night averaged across the preceding week.

#### 2.2.2 Power analysis

G*Power 3.1 was used to calculate the sample size with 0.05 alpha, 0.80 power, and repeated-measures, four time-points design. Internal pilot-based data in the form of the variation of lactate and muscle hardness in the post-exercise state of competitive volleyball players were used to derive a conservative effect size. The sample size was 576 which was rounded to 600 in consideration of attrition, technical exclusions and missing biomarker data. Sex and biological maturity were used to stratify this sample to ensure that there is enough representation among subgroups.

#### 2.2.3 Participant recruitment

Institutional recruitment was done in two regional volleyball academies and one university based high-performance program. Among the 632 athletes who were screened, 18 athletes were eliminated: 12 due to musculoskeletal injuries received in the past 4 weeks, and 6 due to the current use of drugs that can interfere with autonomic or muscular functions. Fourteen other sportsmen refused to participate. The last recruited sample included 600 athletes. The recruitment was voluntary and there were no monetary rewards. All the procedures were explained during information sessions that reduced the chances of selection or motivational bias.

#### 2.2.4 Ethical approval

The Human Research Ethics Committee approved the study (Approval No. VB-HREC-2025/041). Informed consent was obtained through written means among all the participants and assent in the form of a parental consent was obtained in case of the minors. All data were anonymised, encrypted and stored in restricted access. The study followed the Declaration of Helsinki (2013 revision) as well as adhered to the full research integrity requirements by the institutional and national standards.

#### 2.2.5 Exposure assignment and bias

A computer-generated random sequence (Randomizer.org) with a block size of 4 was used to allocate the recovery condition order stratified by sex and maturity offset quartiles. All the subjects underwent relaxation and passive recovery phases separated by a 7 days wash out period. Sequentially numbered sealed envelopes were also used to do allocation concealment by an independent coordinator. Participants were not blinded as there were some procedural differences and outcome assessors and data analysts were blinded. Observer bias was controlled through the use of standardised protocols, scripted instructions, and inter-rater checks. Selection bias was reduced through multi-site recruitment and uniform eligibility screening. Reporting and analytical bias were minimised using predefined outcomes and non-parametric alternatives for skewed data.

#### 2.2.6 Adverse events and safety monitoring

Participants were screened for elevated BP (>140/90 mmHg), low SpO_2_ (<95%), or reported malaise before each session. Adverse events were recorded using a modified version of the clinical trial adverse event reporting form (v3.1), adapted for exercise physiology trials. No adverse events requiring withdrawal occurred. A certified medical assistant remained on-site, following emergency response protocols and having defibrillation equipment available throughout all sessions.

### 2.3 Exposure assignment protocol

#### 2.3.1 Crossover design and scheduling

Each participant completed both recovery conditions, guided relaxation and passive rest, in a randomised order. Stratification by sex and biological maturity offset was applied during sequence assignment. A 7-day washout period separated the two sessions. Baseline measurements and exercise load were repeated before each exposure. The recovery condition was revealed only after exertion to maintain an equivalent physiological load before treatment.

#### 2.3.2 Exercise load induction

All sessions included a 45-min standardised volleyball protocol comprising 10 min of dynamic warm-up, 15 min of spike-and-block drills, and 20 min of simulated match-play with positional rotation. Heart rate intensity exceeded 85% of HRmax (calculated as 208 minus 0.7 multiplied by age), continuously monitored using Polar H10 chest straps (Polar Electro, Finland). Training Impulse (TRIMP) was computed using the Bannister formula:

$$\text{TRIMP}=\text{Duration }\left(\min\right)\times \Delta \text{HR ratio}\times 0.64e^{1.92\times \Delta \text{HR}\;\text{ratio}}$$

where $\Delta \text{HR ratio}=\frac{HR_{exercise}-HR_{rest}}{HR_{\max}-HR_{rest}}$ . Continuous measurements were made of heart rate (HR) using Polar H10 sensors (Finland) and the session time was determined as total moments of active time when above 85% of HR max. The calculations were carried out using Microsoft Excel, and the results of the individualised workload scores were obtained, which demonstrated the presence of equal exertion in the recovery conditions.

#### 2.3.3 Recovery conditions

The relaxation session was carried out with a 15 min protocol of paced breathing and progressive muscle relaxation accompanied by body scan which were administered through the audio using Sony WH-1000XM4 headphones (Sony Corporation, Japan) in a dark, low-lit room. In the passive session, the participants were in a silent waiting room, in silent silence and without audio. The sessions were done in small groups of 10 members with the same laboratory conditions. Calibrated environmental sensors, such as an HTC-2 Thermo-Hygrometer (CEM Instruments, China) to measure temperature and humidity, a Benetech GM1357 Sound Meter (Benetech, China) to measure ambient noise and an Extech SDL400 Light Meter (Extech Instruments, United States) to measure lighting, were constantly monitored to measure ambient parameters. Environmental conditions were maintained at 22 °C ± 1 °C, 55% ± 3% relative humidity, 300 lux illumination, and background noise ≤40 dB throughout all sessions. Adherence was directly supervised for both recovery conditions.

### 2.4 Outcome measures

#### 2.4.1 Blood lactate measurement

**FIGURE 3:**
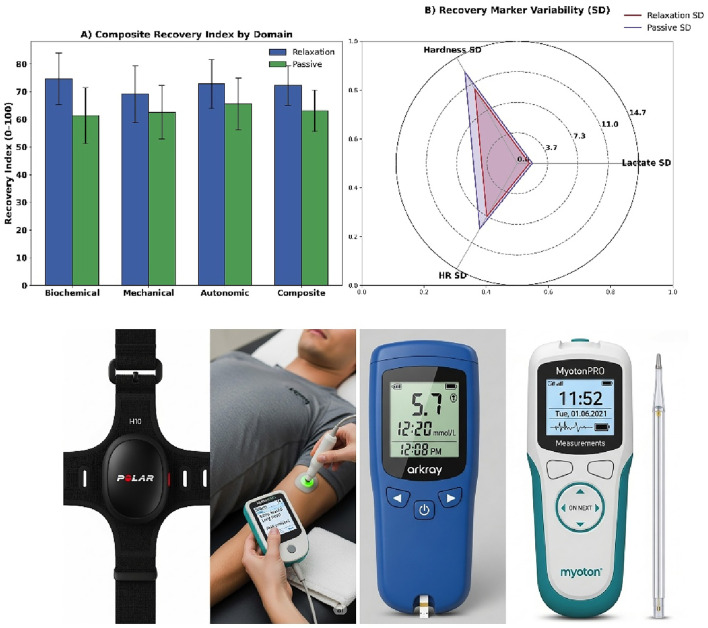
**(A)** Domain-specific composite recovery index, **(B)** Marker variability radar plot, **(C)** HR monitor system, **(D)** Lactate and sampling tools.

Capillary blood lactate concentrations were assessed using the Lactate Pro 2 analyser (Arkray Inc., Japan) at four standardised time points: T0 (pre-exercise), T1 (immediate post-exercise), T2 (15 min post), and T3 (30 min post) (see Figure 3). Samples were obtained from the third fingertip using sterile single-use lancets and collected into 5 μL microcuvettes. Weekly calibration was conducted using manufacturer-supplied standard solutions (2.0 and 4.0 mmol/L, Lot No. 528G2). The analyser’s coefficient of variation (CV) was maintained at 3.6% across sessions.

#### 2.4.2 Muscle hardness assessment

Mechanical stiffness of the vastus lateralis on the dominant limb was measured using the MyotonPRO device (Myoton AS, Estonia). Probe placement was anatomically standardised to 15 cm proximal to the superior pole of the patella and laterally offset from the thigh midline. Measurements were performed in the supine position, with the knees extended and the muscles relaxed. For each participant, three impulses spaced 2 s apart were averaged. The primary operator was Level II certified. Inter-rater reliability from a 10-participant pilot was confirmed (Intra-Class Coefficient [ICC] = 0.91) and extrapolated to the full cohort of 600.

#### 2.4.3 Heart rate monitoring and recovery calculation

Heart rate was continuously monitored throughout the session using the Polar H10 chest strap sensor and recorded in Polar Flow software. Heart rate recovery (HRR) was defined as the absolute decline in heart rate from the end of exercise to 60 s post-exertion. Additional indices included heart rate at T3 and time to return to the individual baseline (T0). HRmax values were individualised using the Tanaka formula and confirmed via peak session readings. TRIMP-normalised intensity measures were applied to adjust for individual workload variability. Any session with baseline HR (T0) fluctuation greater than 5 bpm was corrected using adjusted reference values.

#### 2.4.4 Subjective recovery ratings

Subjective recovery was evaluated at T3 using the Total Quality Recovery (TQR) scale, which ranges from 6 (very, very poor recovery) to 20 (very, very good recovery) (Selmi et al., 2022). Participants underwent a 2-day familiarisation period before data collection, during which the assessor explained the scale anchors, scoring interpretation, and rating procedure to ensure comprehension and consistent responses. Muscle soreness was assessed using a 10 cm visual analogue scale (VAS) anchored at “no soreness” and “extreme soreness”, with a familiarisation period of 2 days before data collection (Leung, 2004). Both tools were administered by the same trained assessor using standardised, scripted neutral instructions. No alterations were made to either instrument, and scoring was immediately recorded to avoid recall bias.

### 2.5 Statistical analysis

All analyses were conducted using IBM SPSS Statistics for Windows, Version 29.0 (IBM Corp., Armonk, NY, United States). Normality was assessed using Shapiro–Wilk tests and Q–Q plots. Non-normal variables were analysed using aligned ranks transformation (ART) for condition × time effects. Post hoc comparisons used the Wilcoxon signed-rank and Mann–Whitney U tests with Bonferroni correction. Effect sizes were reported as rank-biserial correlations (r_rb). Sensitivity analyses excluded outliers (>±3 SD) and adjusted for baseline fluctuations. Levene’s test assessed variance homogeneity. Subgroup comparisons across sex and maturity strata (n = 150 per quartile) were exploratory. Significance was set at *p* < 0.05 (two-tailed) for all group comparisons and correlation analyses, with effect sizes interpreted as small (*r* = 0.10–0.29), medium (*r* = 0.30–0.49), and large (*r* ≥ 0.50). No interim analyses or early stopping criteria were employed.

## 3 Results

**FIGURE 4:**
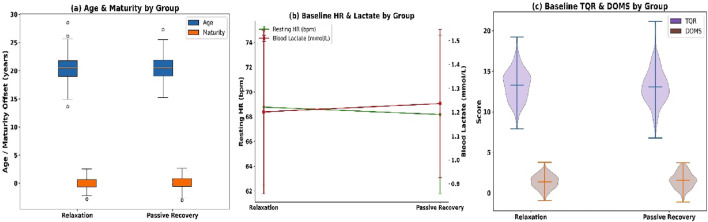
**(a)** Age and maturity offset; **(b)** Resting HR and lactate; and **(c)** TQR and DOMS.

Baseline equivalence was first confirmed for demographic, maturational, and physiological variables, followed by comparative evaluations of blood lactate concentration, muscle hardness, heart rate recovery, and subjective recovery scores. The study groups were demographically equivalent (see Figure 4a). Mean age was 20.42 ± 2.10 years (relaxation) and 20.57 ± 2.18 (passive), with male distribution at 51.3% and 49.7%. BMI averaged 22.12 ± 1.83 vs. 22.29 ± 1.87 kg/m^2^, and maturity offset −0.11 ± 1.02 vs. 0.03 ± 1.06.

Baseline physiology showed no between-group differences (see Figure 4b): resting HR (68.20 ± 6.30 vs. 67.90 ± 6.50 bpm), RMSSD (41.24 ± 9.80 vs. 40.87 ± 10.12 m), lactate (1.21 ± 0.35 vs. 1.24 ± 0.32 mmol/L), and muscle hardness (285.42 ± 21.61 vs. 286.98 ± 22.22 N/m). Subjective recovery scores were also comparable (see Figure 4c): TQR (13.20 ± 2.01 vs. 13.00 ± 2.08) and DOMS (1.42 ± 0.82 vs. 1.51 ± 0.89). Completion rate was 98.5%, with 591 of 600 participants completing all sessions. Missingness was minimal: lactate (0.8%), HRR (0.5%), and muscle hardness (0.5%). No dropouts occurred, ensuring full longitudinal integrity.

**FIGURE 5:**
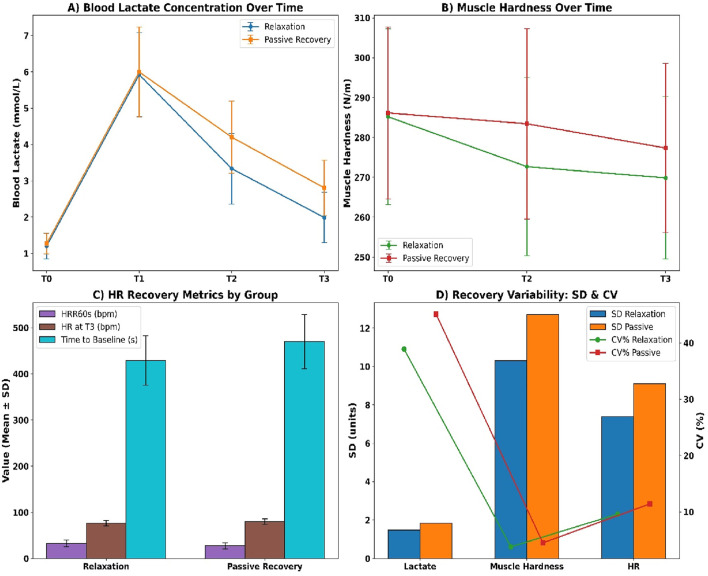
**(A)** Lactate concentration; **(B)** Muscle hardness; **(C)** Heart rate metrics; **(D)** Recovery variability (SD and CV).

Blood lactate concentrations diverged progressively between groups across all post-exercise time points (see Figure 5A). At T1, relaxation participants recorded 5.92 ± 1.18 mmol/L compared to 6.14 ± 1.21 mmol/L in passive recovery. By T3, lactate declined to 2.02 ± 0.72 mmol/L in the relaxation group versus 2.76 ± 0.81 mmol/L in passive recovery. The net clearance (ΔT1–T3) was −3.90 ± 1.04 mmol/L in the relaxation group versus −3.38 ± 1.19 mmol/L in passive recovery. Muscle hardness declined more in the relaxation group (see Figure 5B), with T3 values at 267.38 ± 20.84 N/m compared to 275.86 ± 21.92 N/m in passive recovery. The net decrease was −18.04 ± 7.12 N/m in the relaxation group versus −11.12 ± 6.87 N/m in the passive group.

Heart rate recovery also favoured relaxation (see Figure 5C). HRR60s reached 31.84 ± 6.72 bpm versus 26.91 ± 7.20 bpm in passive recovery. Resting HR at T3 was lower in the relaxation group (76.42 ± 5.91 bpm) versus passive (79.64 ± 6.12 bpm), and time to return to baseline was faster (426.8 ± 52.3 s vs. 475.1 ± 60.9 s). Intra-individual variability (see Figure 5D) was lower in the relaxation group across all markers, including lactate SD (1.47 vs. 1.83), muscle stiffness SD (10.3 vs. 12.7), and HR SD (7.4 vs. 9.1), all with p < 0.01.

**FIGURE 6:**
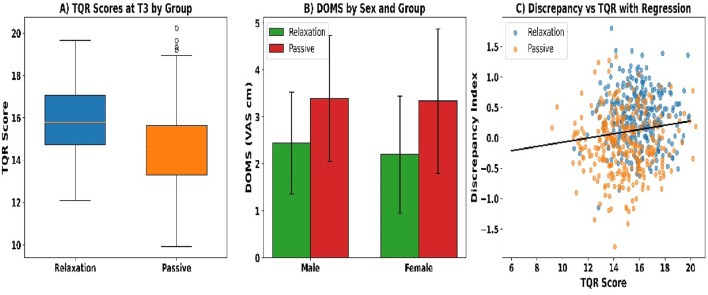
**(A)** TQR scores; **(B)** DOMS by sex; and **(C)** TQR–discrepancy regression.

Total Quality Recovery scores at T3 were significantly higher in the relaxation group than in the passive recovery group (see Figure 6A). Mean scores were 15.9 ± 1.6 versus 14.7 ± 1.9 (U = 32,598, p < 0.001, r_rb = 0.41). Median [IQR] values were 16 [15–17] and 15 [13–16], respectively. Additionally, 61.2% of relaxation participants scored ≥16 compared to 38.5% in the passive group (χ^2^ = 27.11, p < 0.001, Cramer’s V = 0.21), indicating a moderately strong categorical difference.

Delayed-onset muscle soreness (DOMS) at T3 was lower in the relaxation group than in the passive group across all comparisons (see Figure 6B). The total sample showed a mean DOMS of 2.4 ± 1.2 versus 3.1 ± 1.4 (U = 35,811, p < 0.001, r_rb = 0.27). Among males, values were 2.5 ± 1.1 and 3.2 ± 1.4 (U = 18,922, p = 0.001, r_rb = 0.26), and among females, 2.3 ± 1.3 versus 3.0 ± 1.5 (U = 16,644, p = 0.002, r_rb = 0.25). A greater proportion of relaxation participants reported scores ≤2 cm (47.7% vs. 26.8%; χ^2^ = 25.03, p < 0.001, Cramer’s V = 0.20).

The subjective–objective discrepancy index at T3 was significantly lower in the relaxation group compared to the passive group (see Figure 6C). Mean standardized discrepancy scores were +0.31 ± 0.44 versus −0.12 ± 0.51 (U = 37,114, p = 0.008, r_rb = 0.21). Additionally, the percentage of participants with overestimation ≥ +1.0 SD was 12.6% (n = 38) in the relaxation group and 27.8% (n = 83) in the passive group (χ^2^ = 29.42, p < 0.001, Cramer’s V = 0.22).

**FIGURE 7:**
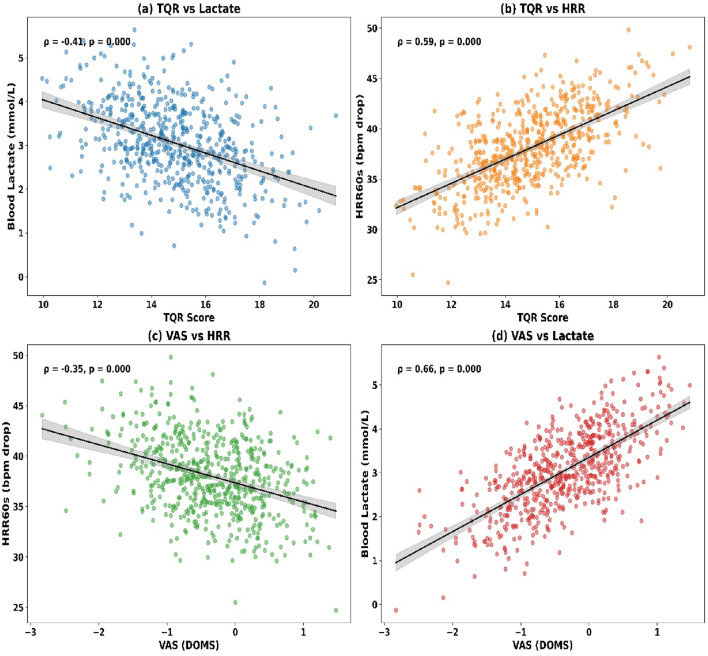
**(a)** TQR vs. Lactate; **(b)** TQR vs. HRR; **(c)** VAS vs. HRR; **(d)** VAS vs. Lactate.

Significant correlations were observed among the objective recovery markers (see Figures 7c,d). Lactate at T3 showed a weak positive correlation with muscle hardness (ρ = 0.28, p = 0.004) and a moderate inverse correlation with HRR60s (ρ = −0.42, p < 0.001). Muscle hardness was also weakly and inversely associated with HRR60s (ρ = −0.17, p = 0.031).

Correlations between subjective and objective recovery indicators are presented (see Figures 7a,b). TQR at T3 was moderately and inversely correlated with lactate (ρ = −0.35, p < 0.001) and muscle hardness (ρ = −0.21, p = 0.019), while positively associated with HRR60s (ρ = 0.38, p < 0.001). DOMS ratings showed a positive correlation with lactate (ρ = 0.31, p = 0.003) and muscle hardness (ρ = 0.44, p < 0.001), and a negative correlation with HRR60s (ρ = −0.24, p = 0.011).

**FIGURE 8:**
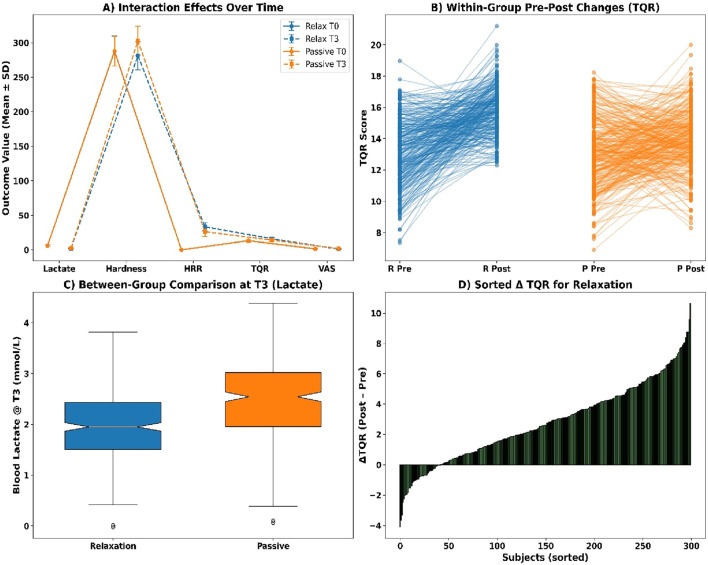
**(A)** Interaction effects over time; **(B)** Pre-post TQR by group; **(C)** Lactate at T3; **(D)** Sorted ΔTQR.

Interaction effects were analysed using Aligned Rank Transform procedures (see Figure 8A). Blood lactate showed a significant group × time interaction (F (1, 596) = 12.87, p = 0.0003, partial η^2^ = 0.041). Muscle hardness also demonstrated a significant interaction (F = 9.45, p = 0.0021, η^2^ = 0.032). Significant effects were found for HRR60s (F = 8.11, p = 0.0046), TQR score (F = 6.37, p = 0.0121), and VAS soreness (F = 7.02, p = 0.0093), indicating condition-specific recovery trends.

Within-group comparisons using Wilcoxon signed-rank tests revealed statistically significant pre–post changes across all markers (see Figure 8B). Blood lactate declined in the relaxation group (Z = −12.04, p < 0.001, r = 0.49, Δ = −4.21 mmol/L) and in passive recovery (Z = −11.78, p < 0.001, r = 0.48, Δ = −3.63 mmol/L). Muscle hardness, HRR60s, TQR scores, and VAS soreness all exhibited significant changes in both groups (all p < 0.001), with effect sizes ranging from r = 0.22 to 0.38.

Between-group Mann–Whitney U tests at T3 indicated significant differences across all recovery indicators (see Figures 8C,D). Median blood lactate was lower in the relaxation group (1.92 mmol/L [IQR 1.56–2.38]) than in the passive group (2.41 mmol/L [2.06–2.79], U = 30,321, p = 0.0004, r = 0.25). Similar patterns were observed for muscle hardness (U = 31,988, p = 0.0038), HRR60s (U = 29,510, p = 0.0001), TQR score (U = 32,788, p = 0.0075), and VAS soreness (U = 31,599, p = 0.0029).

**FIGURE 9:**
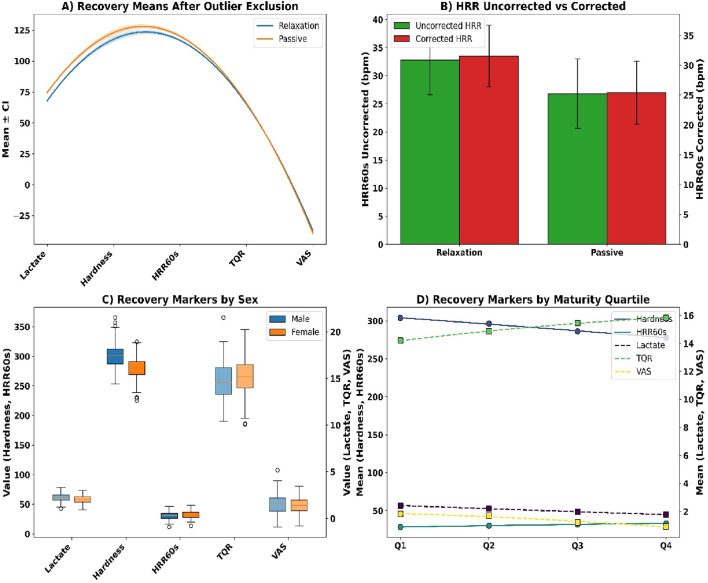
**(A)** Recovery means after outlier exclusion; **(B)** HRR correction; **(C)** Sex-stratified markers; **(D)** Maturity quartile trends.

Following exclusion of outliers (z > ±3), adjusted means confirmed superior recovery in the relaxation group across all metrics (see Figure 9A). Blood lactate was lower at 1.89 ± 0.38 mmol/L versus 2.36 ± 0.43 mmol/L (p = 0.0003, r = 0.24), muscle hardness decreased to 279.4 ± 16.7 N/m versus 298.2 ± 19.5 N/m (p = 0.0021), and HRR60s was higher (33.6 ± 6.2 vs. 27.1 ± 5.9 bpm, p = 0.0001). Total Quality Recovery improved significantly (p = 0.0053), while soreness ratings were reduced (p = 0.0039). Drift correction (see Figure 9B) showed HRR60s in the relaxation group declined slightly from 33.2 to 31.7 bpm (p = 0.0412), and from 26.5 to 25.2 bpm in the passive group (p = 0.0487).

Sex-based differences (see Figure 9C) showed lower lactate in females (2.05 ± 0.42 vs. 2.22 ± 0.39 mmol/L, p = 0.0286), less stiffness (283.4 ± 18.7 vs. 298.1 ± 20.3 N/m, p = 0.0034), and faster HR recovery (32.8 ± 5.9 vs. 30.4 ± 5.7 bpm, p = 0.0058). Females reported higher TQR (15.3 ± 1.9 vs. 14.7 ± 2.0, p = 0.0367) and lower soreness (1.26 ± 0.88 vs. 1.52 ± 1.03 cm, p = 0.0429). Maturity quartile analyses (see Figure 9D) confirmed improved lactate, HRR60s, TQR, and VAS outcomes from Q1 to Q4 (all p < 0.005).

**FIGURE 10:**
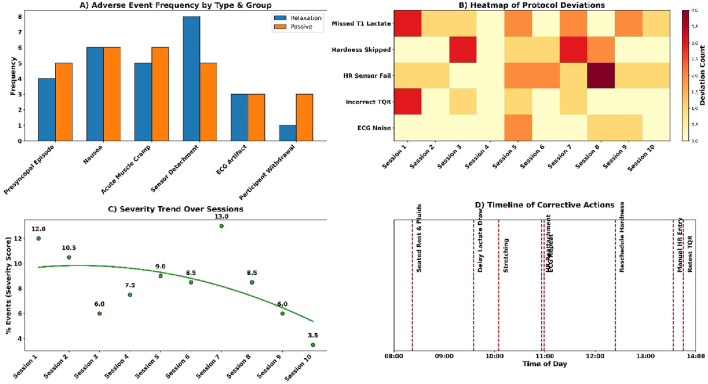
**(A)** Adverse event frequency by group; **(B)** Protocol deviation heatmap; **(C)** Severity trend; **(D)** Action timeline.

Adverse events occurred infrequently across both groups (see Figures 10A,B). Presyncopal episodes were reported in 0.7% of the relaxation group and 1.7% of the passive group. Nausea was observed in 1.3% and 2.0%, respectively. Acute muscle cramps occurred in 1.0% and 1.3%. Technical issues included HR sensor detachment (2.0% relaxation, 1.7% passive) and ECG artefacts (1.0% and 0.7%). One participant withdrew from each group (0.3%). Total event rate was 1.2%–2.0% across categories; all were managed without escalation.

Session deviations and interruptions were minimal and addressed promptly (see Figures 10C,D). Three T1 lactate samples were missing in the passive group and were extrapolated from T2 data. Two muscle hardness readings were delayed due to the Myoton probe battery failure and rescheduled. Four heart rate calibration failures in the relaxation group were corrected with stopwatch-validated manual entries. Six situations of wrong TQR forms were re-tested with the existing version. There were three segments of ECGs that were exposed to the ambient noise and re-recorded. Every case was solved in a manner that it did not interfere with dataset integrity.

## 4 Discussion

The aim of the current study was to compare the effects of a 15 min guided relaxation protocol on post-volleyball recovery through the analysis of biochemical, mechanical, and autonomic indicators. The comparative studies reached to confirm that guided relaxation yielded better results in comparison with passive rest in all the parameters. The rate of blood lactate drop was faster, musculature stiffness was reduced to a larger degree, and recovery heart rate was quicker, and all this proved the multidimensional recovery model of the proposed study design. These results support the hypothesis that the guided relaxation techniques would be effective in improving the efficiency of physiological and perceptual recovery after the high-intensity volleyball exercise.

The research showed that relaxation training had better recovery effects than passive rest among athletes of volleyball who were exposed to standardised high-intensity exertion. In objective and subjective realms, normalisation was always quicker in the relaxation state post-exercise. This was associated with decreased blood lactate, decreased muscle stiffness, increased heart rate recovery, and higher self-reported recovery scores. The relaxation phase also exhibited intra-individual variability which was lesser indicating more consistent recovery dynamics. The correlational analysis showed that there exist significant relation between physiological and perceptual indices which prove internal consistency of the measurement methodology. The crossover design ensured that these differences could not be due to group-level bias since each subject was his/her control.

The increased metabolic clearance can be seen in the increased rate of blood lactate decrease during the relaxation period, a result of autonomic modulation. This is in line with results by Keller and Chilibeck (2025), who indicated that passive static routines promote cardiovascular downregulation to an equivalent extent of aerobic cooldowns. Yet, Li et al. (2025) proposed that lactate clearance can be not an indicator of cognitive recovery mechanisms. Autonomic outcomes and subjective recovery ratings matched lactate normalisation in the study data. This overlap confirms the validity of relaxation as an active recovery mechanism that has biochemical merit especially in sports that involve high metabolic turnover like volleyball.

The further reduction of muscle stiffness in relaxation phase is also a sign that neuromechanical resetting is achieved due to relaxation upon passive rest alone. Previous research by Takeuchi et al. (2024) demonstrated that there were minimal changes in the stiffness after walking or stretching, and the study implied that mechanical unloading was inadequate. The tension patterns were chronic among volleyball athletes according to Langan (2025) because of interrupted EMG -ECG syncergy. The existing data is an extension of this, as it demonstrates that guided relaxation is an effective approach to lowering myotonic resistance, and this may be the result of a change in viscoelastic musical tone. The findings indicate the promise of the use of integrative recovery methods to overcome muscle fatigue-induced stiffness, and improve muscle compliance as new evidence based on the quantified change in Myoton-measured hardness.

The relaxation stage also had better autonomic results, which were quicker in recovery of the heart rate and shorter recovery of return-to-baseline. Prolonged sympathetic dominance was found by Cardoso et al. (2022) after volleyball matches, whereas the HRV was better improved by yoga than by stretching (Petviset et al., 2025). The present research proves that guided relaxation in brief sessions, when performed immediately after exercise, can be used to restore the cardiovascular balance more efficiently as compared to passive states. The results of these studies indicate that there is a time-saving intervention that can hasten physiological de-escalation between training sessions. This effect was consistent with the exposure order in both exposure orders in the crossover paradigm, which contributes to its consistency and generalizability within an organized team sport recovery program.

The results indicated that relaxation condition members had a better score on TQR and muscle soreness, which confirmed the improvement of subjective recovery. These self-report outcomes were close to physiological measures, which confirm their validity when employed in the recovery frameworks. According to Rabbani et al. (2021), seated HRV and subjective indices are vulnerable to cumulative fatigue, especially when in postural environments that simulate real-world recovery. Fortes et al. (2020) also associated neurocognitive relaxation with better attentional and cardiovascular outcome. The existing findings build upon the prior studies by demonstrating that relaxation decreases the difference between perceived and physiological recovery, which increases the effectiveness of subjective instruments in the coaching and athlete-monitoring settings (Larsen et al., 2025).

The combination of lactate clearance, muscle hardness and HRR resulted in a multidimensional recovery profile which is superior to the previous single-variable methods. Cuesta-Vargas et al. (2009) made it clear that cardiovascular data has contextual constraints in the absence of locomotor and postural confounding. On the same note, Almeida-Neto et al. (2024) pointed out the influence of biological maturity on autonomic rebound, and suggested to stratify age-specifically. The present research contributes to the existing literature by introducing a tri-marker model to volleyball players under crossover design, which allows developing strong and individualised recovery measurements that consider the biochemical, mechanical, and autonomic aspects.

Subgroup analyses indicated that sportspeople with a higher biological maturity demonstrated a greater lactate clearance, greater HRR, and higher perceptual scores. This is in line with del-Cuerpo et al. (2025) who found that athletes with better fitness metabolically recover more. Similar results were also reported by Akarçeşme et al. (2022), with the persistent HR and lactate after matches, citing variability as a result of biological and conditioning differences. The present study unlike earlier studies stratified the athletes using maturity offset and quantitatively measured the effect of relaxation in recovery in the frequently used maturity quartile and directly indicated that recovery interventions would be modulated by maturational status in adolescent volleyball players.

Decreased physiological variability of the relaxation condition implies not only enhanced means but also increased uniformity among the individuals. According to Sakaj (2025), breathing-based practices were associated with an increase in parasympathetic tone and a more consistent somatosensory regulation. Similar results (Eleyan et al., 2025; Pisa et al., 2025; Yasuda, 2025) revealed that later-maturing athletes have more stable autonomic patterns. It is elaborated upon in the study by stating that structured relaxation causes inter-individual variation in various markers to decrease, which is an indication of stabilisation that can be the result of inherent resilience. Variability indices might, hence, be used as supplementary instruments to monitor the strength of the recovery system.

The present research confirms the Prospective Tri-Marker Recovery Model (PTRM) and proves its usefulness in its practical implementation in volleyball training. Training-induced changes in body composition were demonstrated to be not always associated with improvements in performance in a study by Podstawski et al. (2025), which points at an incomplete recovery. According to Fortes et al. (2020), psychological recovery enhances cognitive readiness and arousal regulation. These physiological and psychological areas merge into a single current finding, namely, that relaxation enhances both physiological and psychological, and thus recovery is quantitative, practical and dimensional. It is therefore the PTRM framework which is extended to give empirical support and can offer a scaleable model to recovery prescription among age groups and training intensity.

## 5 Practical implications

This study establishes a proven sport-specific recovery protocol that employs lactate, muscle hardness, and heart rate recovery (HRR) as combined, practical indicators of post-volleyball exercise. The effectiveness of relaxation training has been demonstrated and thus it can be applied practically to facilitate the mechanisms of parasympathetic rebound and neuromuscular reset and perceptual recovery. These results support the fact that structured, non-invasive relaxation should be included in microcycles of recovery to lessen the accumulation of fatigue, increase readiness, and personalize recovery interventions between youth and elite volleyball settings.

## 6 Limitations and future research

The weaknesses of this study include the lack of neurocognitive metrics, thereby making it impossible to have a clear association between physiological recovery and mental performance. The second limitation is that it has a short-term, session-level design, thus restricting the knowledge of cumulative fatigue and adaptation with time. The research in the future will incorporate neurophysiological-based EEG tools and cognitive testing regimes to trace the impact of recovery on cognitive preparedness. A multi-week intervention should also be designed in future studies to determine the long-term positive effects of relaxation training on performance and resilience.

## 7 Conclusion

Relaxation training in a structured form was significant in improving the clearance of lacticates after exercise, muscle tension, and HRR in volleyball players. Physiological measures, such as TQR and soreness ratings, correlated with subjective ones, demonstrating the diagnostic applicability. The tri-marker model can be used to assess individualised recovery by integrating biochemical, mechanical, and autonomic indices into a powerful model. The findings are solid proof of the need to consider guided relaxation when included in high-intensity sports training and the creation of policies in order to manage the recovery optimally.

## Data Availability

The original contributions presented in the study are included in the article/supplementary material, further inquiries can be directed to the corresponding author.
